# Whole-Genome Sequencing Reveals Differences among Kingella kingae Strains from Carriers and Patients with Invasive Infections

**DOI:** 10.1128/spectrum.03895-22

**Published:** 2023-05-17

**Authors:** Omer Murik, David A. Zeevi, Tzvia Mann, Livnat Kashat, Marc V. Assous, Orli Megged, Pablo Yagupsky

**Affiliations:** a Translational Genomics Laboratory, Medical Genetics Institute, Shaare Zedek Medical Center, Jerusalem, Israel; b Microbiology Laboratory, Shaare Zedek Medical Center, Jerusalem, Israel; c Faculty of Medicine, Hebrew University of Jerusalem, Jerusalem, Israel; d Pediatric Department and Infectious Diseases Unit, Shaare Zedek Medical Center, Jerusalem, Israel; e Clinical Microbiology Laboratory, Ben-Gurion University of the Negev, Beer-Sheva, Israel; Taichung Veterans General Hospital; National Chung Cheng University

**Keywords:** *Kingella kingae*, carriage, invasive diseases, pangenome, virulence determinants, whole-genome sequencing

## Abstract

As a result of the increasing use of sensitive nucleic acid amplification tests, Kingella kingae is being recognized as a common pathogen of early childhood, causing medical conditions ranging from asymptomatic oropharyngeal colonization to bacteremia, osteoarthritis, and life-threatening endocarditis. However, the genomic determinants associated with the different clinical outcomes are unknown. Employing whole-genome sequencing, we studied 125 international K. kingae isolates derived from 23 healthy carriers and 102 patients with invasive infections, including bacteremia (*n* = 23), osteoarthritis (*n* = 61), and endocarditis (*n* = 18). We compared their genomic structures and contents to identify genomic determinants associated with the different clinical conditions. The mean genome size of the strains was 2,024,228 bp, and the pangenome comprised 4,026 predicted genes, of which 1,460 (36.3%) were core genes shared by >99% of the isolates. No single gene discriminated between carried and invasive strains; however, 43 genes were significantly more frequent in invasive isolates, compared to asymptomatically carried organisms, and a few showed a significant differential distribution among isolates from skeletal system infections, bacteremia, and endocarditis. The gene encoding the iron-regulated protein FrpC was uniformly absent in all 18 endocarditis-associated strains but was present in one-third of other invasive isolates. Similar to other members of the *Neisseriaceae* family, the K. kingae differences in invasiveness and tropism for specific body tissues appear to depend on combinations of multiple virulence-associated determinants that are widely distributed throughout the genome. The potential role of the absence of the FrpC protein in the pathogenesis of endocardial invasion deserves further investigation.

**IMPORTANCE** The wide range of clinical severities exhibited by invasive Kingella kingae infections strongly suggests that isolates differ in their genomic contents, and strains associated with life-threatening endocarditis may harbor distinct genomic determinants that result in cardiac tropism and severe tissue damage. The results of the present study show that no single gene discriminated between asymptomatically carried isolates and invasive strains. However, 43 putative genes were significantly more frequent among invasive isolates than among pharyngeal colonizers. In addition, several genes displayed a significant differential distribution among isolates from bacteremia, skeletal system infections, and endocarditis, suggesting that the virulence and tissue tropism of K. kingae are multifactorial and polygenic, depending on changes in the allele content and genomic organization. Further analysis of these putative genes may identify genomic determinants of the invasiveness of K. kingae and its affinity for specific body tissues and potential targets for a future protective vaccine.

## INTRODUCTION

The increasing use of nucleic acid amplification assays has resulted in the recognition of Kingella kingae as the leading cause of osteoarthritis and a frequent agent of bacteremia in children 6 months to 4 years of age (1, 2). Kingella kingae also causes endocarditis in children and adults (3). Similar to other members of the *Neisseriaceae* family, K. kingae is asymptomatically carried on the oropharynx (4), and the colonized mucosa is the source of droplet transmission through close physical contact between young children and of the entry of K. kingae into the bloodstream, from which it disseminates to distant sites (5–7).

Skeletal system infections constitute 55% of all cases of invasive K. kingae disease, followed by occult bacteremia in 39% of cases, whereas endocarditis is uncommon, representing <2% of all clinical infections (2). Kingella kingae nonendocardial infections (NEIs) affect almost exclusively young children, are characterized by mild local and systemic inflammation, and, if adequately treated with antibiotics, have an excellent prognosis, leaving no long-term disabilities (1, 2, 8–10). In contrast, patients with K. kingae endocarditis have high fever and elevated acute inflammation markers (2, 10, 11). Despite the susceptibility of K. kingae to β-lactam and aminoglycoside antibiotics that are usually administered to endocarditis patients (12), this pathogen causes rapid destruction of the cardiac valves, and the resulting friable vegetations entail an increasing risk of life-threatening embolic phenomena in the brain and peripheral arteries (2, 3, 11). Patients with K. kingae endocarditis that is unresponsive to medical treatment require emergency cardiac surgery, the mortality rate is high, and many patients later need valve correction or replacement (2, 3, 11). The striking difference between the severe features of K. kingae endocarditis and the benign clinical course of NEI suggests that strains that invade the endocardium may harbor distinct genomic determinants that result in cardiac tropism, a more robust inflammatory response, and extensive tissue damage. However, despite the increasing appreciation of K. kingae as an important human pathogen, the species’ population structure and the genomic determinants associated with the different clinical outcomes remain largely unknown. To the best of our knowledge, the full genomes of only 6 isolates have been published, and the draft assemblies of another 51 isolates have been deposited in public databases (https://www.ncbi.nlm.nih.gov/assembly). Employing whole-genome sequencing (WGS), a study was conducted to analyze the population structure of the species and to identify genes associated with asymptomatic carriage and invasive infections. The study results might contribute to identifying novel targets for a future protective vaccine.

## RESULTS

### Origin of the strains included in the study.

Twenty-three isolates from unrelated asymptomatic carriers, 23 from patients with bacteremia without focal infection, 61 from children with joint or bone infections, and 18 from children and adult patients with endocarditis were studied. Strains were isolated in Israel (*n* = 91), France (*n* = 16), Canada (*n* = 8), Spain (*n* = 6), Norway (*n* = 2), the United States (*n* = 1), and Russia (*n* = 1). A detailed description of these 125 strains is provided in Table 1.

**TABLE 1:** Demographic, clinical, and MLST characteristics of the 125 K. kingae strains included in the study

Isolate name	Isolation conditions			Clinical condition				ST	STC
	Year	Country	City/region	Carriage (*n* = 23)	Skeletal infection (*n* = 61)	Bacteremia (*n* = 23)	Endocarditis (*n* = 18)		
1699	2014	Israel	Jerusalem		+			71	
9001970	2012	Israel	Center		+			24	23
1662-A6845	2015	Israel	Jerusalem				+	24	23
35-A372	2010	Israel	Jerusalem				+	21	23
ATCC 23330	1966	Norway	Oslo	+				1	1
ATCC 23331	1960s	USA	Unknown			+		23	23
ATCC 23332	1960s	Norway	Oslo			+		17	14
AUD 31930140-S	2013	France	Paris		+			56	23
B0458	2017	Israel	Jerusalem		+			22	23
B0605	2019	Israel	Jerusalem		+			75	
B0802	2016	Israel	Jerusalem		+			72	14
B0812	2019	Israel	Jerusalem		+			6	6
B10615	2008	Israel	Center		+			11	11
B1389	2018	Israel	Jerusalem			+		72	14
B1821	2015	Israel	Jerusalem		+			6	6
B3212	2018	Israel	Jerusalem				+	25	23
B3756	2017	Israel	Jerusalem		+			75	
B3892	2017	Israel	Jerusalem		+			75	
B4104	2019	Israel	Jerusalem		+			73	14
B5743	2019	Israel	Jerusalem			+		73	14
B6249	2021	Israel	Jerusalem				+	75	
B7142	2017	Israel	Jerusalem		+			76	
B7216	2018	Israel	Jerusalem		+			73	14
B7595	2016	Israel	Jerusalem		+			6	6
B7600	2018	Israel	Jerusalem		+			71	
B8255	2019	Israel	Jerusalem			+		21	23
B8907	2018	Israel	Jerusalem				+	73	14
B9852	2017	Israel	Jerusalem		+			75	
BB11960	2004	Israel	Center		+			35	35
BOU 30672	2010	France	Paris		+			14	14
CA105	2009	Spain	Catalonia			+		62	23
CA138	2012	Spain	Catalonia		+			14	14
CA139	2012	Spain	Catalonia	+				63	
CA20	1998	Spain	Catalonia		+			23	23
CA64	2004	Spain	Catalonia			+		6	6
CA77	2006	Spain	Catalonia		+			25	23
CA99	2009	Spain	Catalonia		+			61	6
CAN1	2003	Canada	Montreal		+			25	23
CAN16	2010	Canada	Montreal		+			58	35
CAN2	2005	Canada	Montreal		+			12	11
CAN21	2012	Canada	Montreal		+			59	6
CAN22	2012	Canada	Unknown			+		6	6
CAN25	2013	Canada	Montreal		+			60	14
CAN8	2007	Canada	Montreal		+			14	14
CAN9	2007	Canada	Montreal		+			62	23
CIP 101722	1985	France	Grenoble			+		14	14
CIP 102473	1986	France	Paris		+			19	
CIP 73.01	1972	France	Unknown			+		14	14
DAG 31560001-S	2013	France	Paris		+			55	23
DER 112012-1	2012	France	Paris		+			42	14
ETI 126580	2011	France	Paris		+			43	
F0228	2019	Israel	Jerusalem		+			75	
F1990_B1887	2017	Israel	Jerusalem		+			6	6
FOF 3022006-S	2013	France	Paris		+			46	6
KK100	1996	Israel	South		+			24	23
KK104	1994	Israel	South	+				23	23
KK113	1996	Israel	South	+				31	34
KK114	1996	Israel	South	+				23	23
KK12	1994	Israel	South	+				14	14
KK120	1996	Israel	South	+				11	11
KK128	1997	Israel	North				+	24	23
KK136	1998	Israel	North			+		14	14
KK138	1998	Israel	North		+			24	23
KK144	1998	Israel	South		+			24	23
KK145	1998	Israel	Jerusalem		+			6	6
KK153	1999	Israel	North				+	23	23
KK154	1999	Israel	South			+		18	14
KK164	2000	Israel	South			+		23	23
KK168	2001	Israel	South		+			48	
KK171	2001	Israel	North		+			5	
KK174	1998	Israel	South			+		29	29
KK180	2002	Israel	Center				+	10	35
KK183	2002	Israel	North		+			13	
KK189	2002	Israel	South		+			35	35
KK190	2002	Israel	South				+	24	23
KK194	2003	Russia	St. Petersburg	+				9	29
KK197	2003	Israel	Center				+	24	23
KK199	2004	Israel	Jerusalem				+	6	6
KK208	2004	Israel	South			+		27	
KK212	2004	Israel	South	+				24	23
KK220	2004	Israel	South		+			51	
KK242	2005	Israel	South			+		27	
KK244	2005	Israel	North			+		7	6
KK245	2005	Israel	South			+		33	34
KK247	2006	Israel	Jerusalem				+	38	34
KK253	2007	Israel	South		+			23	23
KK263	2008	Israel	Center			+		7	6
KK267	2008	Israel	South		+			53	
KK3	1994	Israel	South	+				5	
KK411	2008	Israel	Jerusalem				+	6	6
KK416	2011	Israel	Jerusalem				+	24	23
KK420	2011	Israel	Center		+			54	
KK433	2012	Israel	Center		+			52	35
KK444	2013	Israel	Center				+	12	11
KK448	2014	Israel	North				+	74	
KK470	2018	Israel	South				+	35	35
KK56	1994	Israel	South		+			11	11
KK60	1994	Israel	South				+	2	1
KK70	1993	Israel	South		+			4	14
KK81	1991	Israel	South		+			22	23
KK83	1991	Israel	South		+			14	14
KK86	1994	Israel	South	+				6	6
KK88	1996	Israel	North		+			2	1
KK92	1996	Israel	North			+		6	6
KK93	1995	Israel	South			+		24	23
KK98	1992	Israel	South			+		22	23
KWG1	2013	France	Paris		+			41	14
MAR_1853	2011	France	Paris		+			32	34
N10-10770	2010	France	Nantes			+		28	29
N10-6318	2010	France	Nantes		+			23	23
NICE476	2012	France	Nice		+			6	6
PER1851	2011	Israel	South	+				31	34
PER251	2010	Israel	South	+				58	35
PER2748	2011	Israel	South	+				28	29
PER3	2010	Israel	South	+				8	
POH14284	2011	France	Paris		+			18	14
SAI11985	2011	France	Paris		+			26	23
Sch1614	2012	Israel	South	+				27	
Sch187	2010	Israel	South	+				3	3
Sch1931	2012	Israel	South	+				35	35
Sch2108	2012	Israel	South	+				24	23
Sch258	2010	Israel	South	+				18	14
Sch429	2011	Israel	South	+				11	11
Sch87	2010	Israel	South	+				49	
ZUL30220039-S	2013	France	Paris	+				50	1
Total				23	61	23	18		

### Genome size and gene content.

Each sequencing library resulted in 0.5 million to 2 million reads, giving a predicted average genome coverage of 30× to 150×. *De novo* assembly of the 125 genomes resulted in a mean genome size of 2,024,228 bp (95% confidence interval, 2,017,811 bp to 2,030,644 bp). The mean sequence length of the shortest contig at 50% of the total genome length (*N*_50_) was 48.8 kb (range, 26 kb to 76 kb). Genome annotations of the *de novo* assemblies predicted an average of 1,964 putative genes per genome (range, 1,836 to 2,091 genes).

### Genomic diversity among the study’s isolates.

**FIG 1:**
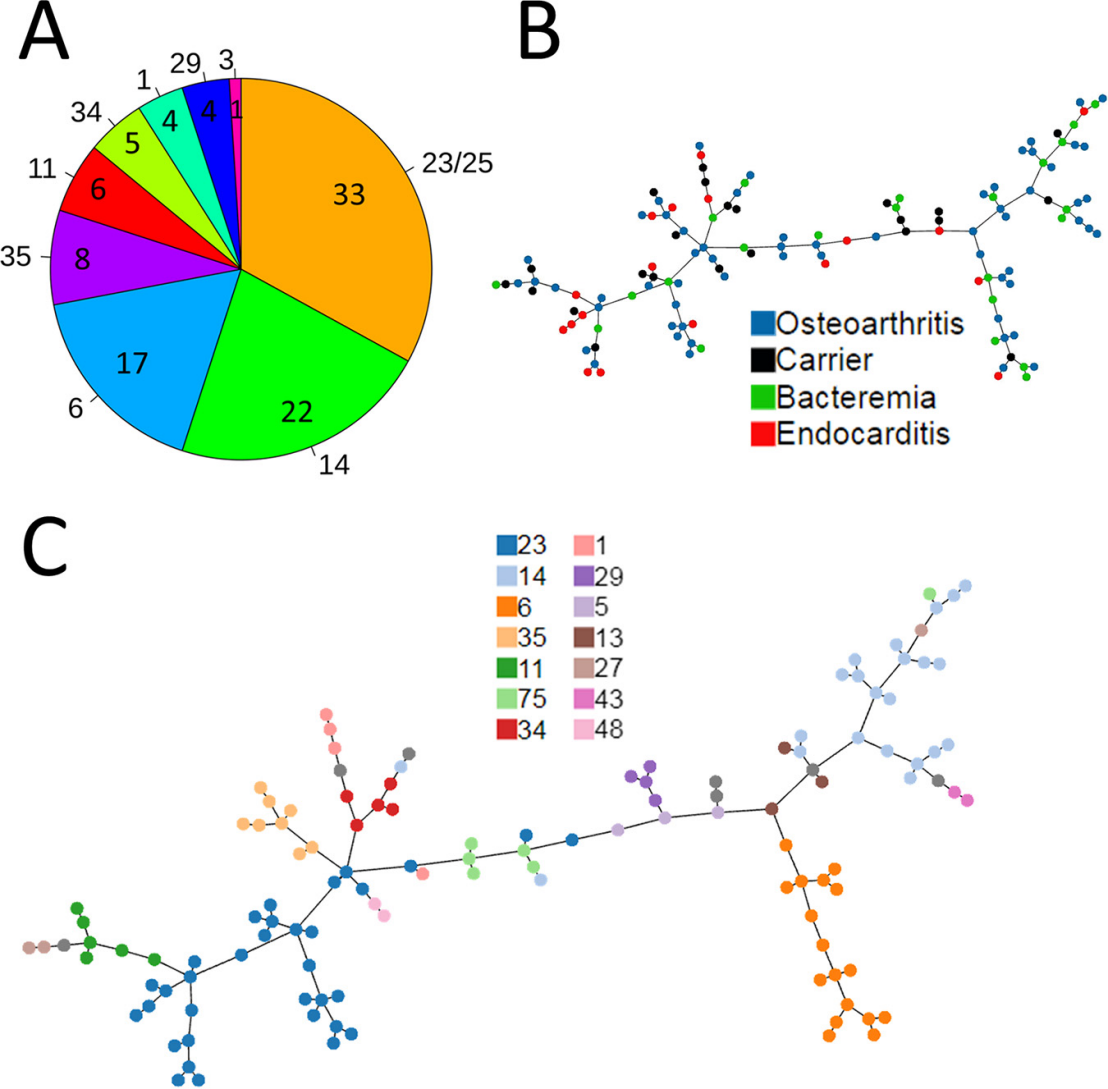
Diversity of the 125 K. kingae isolates analyzed in this study. (A) Pie chart based on the number of isolates belonging to the indicated MLST STC. (B) PHYLOVIZ visualization of the cgMLST of all 125 isolates, color-coded according to the clinical condition. (C) Same as in panel B, color-coded for MLST.

Fifty-five distinct multilocus sequence typing (MLST) sequence types (STs) belonging to 9 ST complexes (STCs) were identified among the 125 sequenced isolates (Fig. 1A). Twenty-five isolates were less similar to other isolates and could not be allocated to a specific STC. The most frequent STs were ST-24 (*n* = 12 isolates), ST-6 (*n* = 12), ST-14 (*n* = 8), ST-23 (*n* = 8), ST-75 (*n* = 6), ST-11 (*n* = 4), ST-73 (*n* = 4), and ST-35 (*n* = 4) (Table 1). New STs were assigned for 15 isolates because their allele content did not match any of the STs deposited in the K. kingae typing data set (http://bigsdb.pasteur.fr/kingella). The novel STs included ST-71 (2 isolates), which belonged to STC-13, ST-72 (*n* = 2) and ST-73 (*n* = 4), both of which belonged to STC-14, and ST-75 (*n* = 6) and ST-76 (*n* = 1), which could not be assigned to a STC. These 15 STs were restricted to the area of Jerusalem, Israel, and were isolated between 2017 and 2021 (Table 1).

To use the power of WGS to increase the genomic diversity resolution, compared to standard MLST, we performed core genome MLST (cgMLST) for the isolates. Allelic diversity for 1,429 single-copy genes shared by all isolates was assessed, resulting in 125 different cgMLST types (Fig. 1B); in order to organize the cgMLST and MLST phylogenies, we color-coded the same scheme according to the MLST ST of each isolate (Fig. 1C). Both MLST and cgMLST revealed that the isolates did not cluster based on the clinical condition. This means that, for example, there was no common phylogenetic ancestor for all isolates cultured from endocarditis patients. Because the visualization of cgMLST results (Fig. 1B and C) is based on allelic differences and lacks information about the number of sequence differences between the genomes, we further performed phylogenetic analysis of the core genome. We used Roary, MAFFT, and FastTree (see Materials and Methods) to produce the core genome maximum likelihood unrooted phylogenetic tree (see Fig. S1 in the supplemental material). The phylogenetic analysis supported the cgMLST results, demonstrating that the ability to infect the endocardium is not related to a specific evolutionary branch. Pairwise analysis of single-nucleotide variations (SNVs) between every two genome assemblies revealed 5 clusters of isolates that had <50 SNV differences, with 2 to 5 isolates in each cluster (see Fig. S2). Since this threshold was never tested in K. kingae, we chose the cutoff value of 50 SNVs based on studies with other bacteria (13). This analysis revealed that our isolates were very diverse and there was no group of clonal isolates that would bias our downstream analyses. Moreover, we observed clusters of high genetic relatedness among isolates cultured from patients with different clinical conditions.

### Core genome and pangenome.

Comparative genomic analysis of all 125 genome assemblies revealed a set of 1,460 core genes detected in all isolates, 85 soft-core genes found in 95 to 99% of the isolates, 814 shell genes found in 15 to 95% of the isolates, and 1,667 cloud genes found in <15% of the isolates. In total, the pangenome consisted of 4,026 predicted genes. Of these, 3,430 (85%) have orthologues according to eggNOG-mapper analysis. As expected, the number of core genes gradually diminished as an increasing number of genomes were studied. However, the value stabilized, and adding more genomes did not result in significant reductions of the core and soft-core sizes (see Fig. S3). To further validate this point, we added the genome assemblies of 47 K. kingae strains deposited in the National Center for Biotechnology Information (NCBI) database. The addition of these genomes, which represented a 37.9% increment in the strain population, increased the pangenome size to 5,393 genes (a 1,367-gene [33.9%] increment) but only reduced the core genome size to 1,393 genes (a decrease of merely 67 genes [4.5%]), indicating that the species exhibits a high degree of genomic conservation.

### Functional genomics of the pangenome.

**FIG 2:**
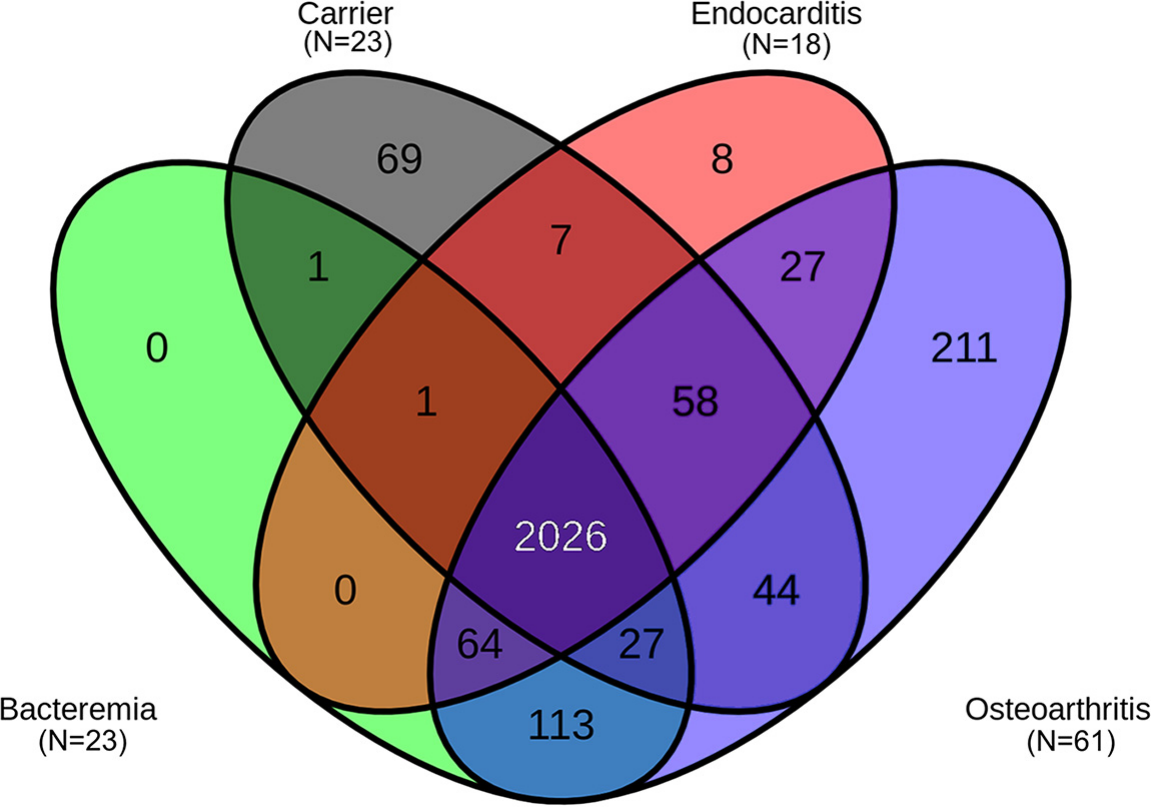
Venn diagram showing the number of genes in the pangenome associated with each of the four clinical conditions, including only genes found in ≥5 isolates.

The presence or absence of the pangenome genes according to the clinical condition is shown in Fig. 2 for genes present in ≥5 isolates. As expected, the vast majority of the genes (*n* = 2,026) were shared. However, several genes were particularly associated with specific conditions. For instance, 8 genes were found in ≥5 of the 18 endocarditis-associated isolates but in ≤4 isolates from the 107 patients with NEI and healthy carriers.

**FIG 3:**
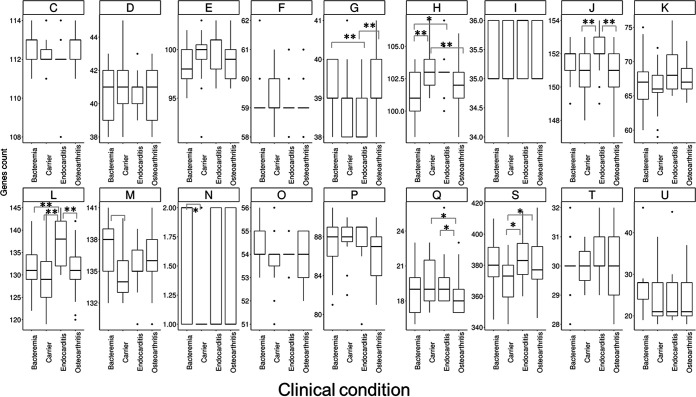
Distribution of the number of genes per isolate associated with each COG category and distributed in the four clinical condition groups. *, *P* < 0.05; **, *P* < 0.01, one-way ANOVA. COG categories are as follows: C, energy production and conversion; D, cell cycle control and mitosis; E, amino acid metabolism and transport; F, nucleotide metabolism and transport; G, carbohydrate metabolism and transport; H, coenzyme metabolism; I, lipid metabolism; J, translation; K, transcription; L, replication and repair; M, cell wall/membrane/envelope biogenesis; N, cell motility; O, posttranslational modification, protein turnover, and chaperone functions; P, inorganic ion transport and metabolism; Q, secondary structure; T, signal transduction; U, intracellular trafficking and secretion; S, unknown function.

The functional group for each protein encoded in the pangenome was determined using the database of the Clusters of Orthologous Genes (COGs). We further investigated the number of genes in each COG for each isolate and grouped the isolates according to their clinical condition (Fig. 3). The numbers of proteins per isolate in COGs G, H, J, L, N, Q, and S (carbohydrate metabolism and transport, coenzyme metabolism, translation, replication and repair, cell motility, secondary structure, and unknown function, respectively) showed statistically significant differences between isolates belonging to the four clinical conditions (one-way analysis of variance [ANOVA] results) (Fig. 3). Interestingly, the mean number of genes from COG L (replication and repair) was significantly higher in endocarditis-associated isolates.

### Genes associated with invasive infections.

To look for genomic features that facilitate deep tissue invasion, we searched for genes that were present in all invasive isolates and absent in colonizers and vice versa. Although no single gene met this criterion, 43 genes were more common among invasive isolates than among carried organisms (see Table S1). None of the genes was significantly enriched after multiple-test correction (Bonferroni-adjusted *P* of <0.05). While 7 of these genes were components of type IV secretion systems and 2 others were related to pilus assembly, the rest were mostly for proteins of unknown function.

### Endocarditis-associated genes.

We could not identify genes whose presence was exclusively associated with bacterial endocarditis. However, 13 genes were found in ≥3 of the 18 endocarditis-causing isolates and were lacking in NEI organisms. Six of those genes are of unknown function, 3 are for transposases, and 2 are for DNA methylases; the remaining 2 are for a signal transduction protein containing a PAS/PAC domain and a 5-oxoprolinase (*pxpB*). Another 14 genes were more common among endocarditis-associated strains than among NEI-associated or carried strains (see Table S2). Six of those genes were significantly enriched after multiple-test correction (Bonferroni-adjusted *P* value of <0.05). Remarkably, the pangenome includes two types of the *frpC* gene, encoding the iron-regulated protein FrpC, one of which was absent in all endocarditis-associated strains and present in 31 (29.0%) of the 107 isolates from carriers and NEI patients and in 27 (32.1%) of the 84 isolates derived from bacteremia or skeletal system infections (*P* values of <0.05 for both comparisons). Further investigation revealed another type of the *frpC* gene that was found in isolates from all clinical conditions.

### Comparison of endocarditis-associated and NEI-associated strains belonging to STC-23/25.

Remarkably, 8 (44.4%) of the 18 endocarditis-associated isolates belonged to STC-23/25, compared to only 25 (23.4%) of 107 non-endocarditis-associated strains (*P* = 0.112 by the chi-square test). Therefore, we looked for genomic differences between endocarditis-associated and non-endocarditis-associated strains within this STC. No particular genes were found in all endocarditis-associated isolates and absent in those derived from patients with other conditions, although 9 genes were overrepresented in STC-23/25 endocarditis-associated isolates, all of them of unknown function (see Table S3). None of them remained significantly enriched after multiple-test correction (Bonferroni-adjusted *P* value of <0.05).

## DISCUSSION

Although the number of healthy carriers of pathogenic bacteria is much larger than the number of individuals with disease, research on the population structure of many bacteria has been hampered by strain collections that are biased toward particularly virulent isolates, often neglecting less invasive organisms and underestimating the extent of genetic diversity of the species (14). In the present study, we selected a large international sample of colonizing and invasive K. kingae strains isolated over a long period to ensure representative genomic diversity. The results show that the genomic content of K. kingae (~2.2 million bp) is in the range of the sizes of other pathogenic *Neisseria* species, such as Neisseria meningitidis and Neisseria gonorrhoeae (https://www.ncbi.nlm.nih.gov/datasets/genome/?taxon=482).

Kingella kingae is naturally transformable, and horizontal gene transfer explains its remarkable genomic heterogeneity (15, 16). To date, 70 distinct MLST profiles have been described for the species (2, 16), and the results of the present study increase the number of known allele combinations to 76. The strain population included many STs belonging to the globally distributed STCs STC-6, -14, -23, -25, and -35 but also STs exhibiting a more restricted geographic dispersion (15, 16) and 5 novel STs limited to the Jerusalem area.

The fact that a given strain was isolated from the oropharynx does not necessarily imply that the organism is unable to cause an invasive infection. All of the strains included in the study had initially colonized the oropharynx, although only those isolated from individuals with disease had also breached the epithelium and entered the bloodstream, causing bacteremia. Of those, a few subsequently invaded the endocardial or skeletal tissues, for which the species exhibits particular affinity. Therefore, although mucosal colonization is a *sine qua non* precondition for the development of clinical disease, not all K. kingae strains are able to penetrate the oropharyngeal epithelial barrier, survive in the bloodstream, and colonize the skeletal system or the endocardium. Previously, a total of 32 distinct K. kingae clones were identified by pulsed-field gel electrophoresis (PFGE) (2), of which 5 (clones B, H, K, N, and P) were isolated in 132 (72.9%) of 181 Israeli patients with disease, indicating increased virulence (17). Remarkably, clones A, C, G, J, M, R, and T, which collectively represented 93 (38.8%) of all pharyngeal isolates from 240 healthy carriers (5), were detected in only 10 (4.4%) invasive infections, suggesting diminished invasive capability (17). Wide differences in virulence among K. kingae strains were also demonstrated in animal models, ranging from an inability to establish infection to a rapidly progressing and fatal illness (16, 18). In the present study, the carried group of isolates probably represented a mixture of organisms with diverse invasive capabilities. This heterogeneity might have blurred the genomic differences between true mere colonizers (i.e., colonizing strains unable to cause disease) and invasive organisms. Despite this potential attenuation, the comparisons demonstrated genomic differences between organisms isolated from the oropharyngeal mucosa and those detected in infected body sites.

The possibility that K. kingae strains also show affinity for specific human tissues is supported by observations of outbreaks of infection in daycare centers (19). These events are naturally occurring quasiexperiments in which a single strain is introduced into a crowded facility attended by a susceptible young population, simultaneously or successively infecting multiple children within a short period (19). This phenomenon offers a unique opportunity to observe the strain’s tropism for specific body sites. Analysis of these clusters has demonstrated that some K. kingae strains tend to cause the same clinical disease, i.e., septic arthritis, osteomyelitis, or tenosynovitis, suggesting tissue specificity, while others invade a variety of body niches, including the endocardium (19–21).

The present study showed that the ability to invade the cardiac valves is widespread among K. kingae strains, and 13 different STs were identified among the 18 endocarditis-associated isolates. However, 8 of the identified STs belonged to the close-knit STC-23/25. It should be pointed out that members of this STC, i.e., strain SAN 38360 (22) and strain COU 1310053120 (15), were recovered from two French children with bacterial endocarditis. STC-23/25 represented 52 (26.5%) of 196 non-endocarditis-associated strains in an international collection of invasive organisms isolated between 1966 and 2014, indicating that this STC is highly virulent, has disseminated worldwide, and remained stable over a long period (15, 16). However, the fraction of cases of endocarditis caused by STC-23/25 organisms in the present study (8 [44.4%] of 18 cases) did not statistically differ from the representation of this STC among NEI strains (15, 16) (*P* = 0.18), suggesting that this STC, although highly invasive, does not exhibit a particular tropism toward the cardiac valves.

Unlike *Enterobacterales* species, among which acquisition of pathogenic islands by horizontal transfer results in strains that cause distinct clinical diseases (23), the genetics of pathogenicity in the *Neisseriaceae* family are more complex, depending on combinations of multiple virulence-associated genes distributed throughout the genome (24). The present study results failed to identify single genes whose presence or absence could clearly separate mere colonizers from invasive strains. Instead, K. kingae organisms associated with bacteremia, bone and joint disease, and endocarditis showed statistically significant differences in the distribution of multiple genes. The observed differences in virulence and tissue tropism between K. kingae isolates may then be multifactorial and polygenic, depending on subtle changes in the allele content and the overall genomic organization (24). Alternatively, it can be argued that the aggressive clinical course observed among patients with K. kingae endocarditis depends not on the biological features of the strain but on the protective microenvironment of cardiac valve vegetations, which is poorly accessible to antibiotics, neutrophils, antibodies, and other host defense molecules (25). This inaccessibility results in bacterial multiplication to a very high density, with consequent tissue destruction, and the disintegration of the friable vegetations causes persistent bacteremia and metastatic embolic phenomena (25).

It should be pointed out, however, that a gene encoding the iron-regulated protein FrpC was uniformly absent in all 18 endocarditis-associated strains but was present in one-third of NEI-associated isolates (another type of FrpC was found in all isolates). Although the role of FrpC in the pathogenesis of K. kingae infections has not yet been determined, the analogous protein of N. meningitidis appears to function as an adhesin to the respiratory epithelium (26). The *frpC* gene is present in all invasive meningococcal strains and the majority of carried meningococcal strains (27), while the species is an exceptional etiology of bacterial endocarditis (28). These observations could lead to the speculation that the K. kingae FrpC protein offers an initial selective advantage, facilitating the colonization of the pharyngeal mucosa, but is detrimental to the invasion of endocardial tissues. Future research is needed to elucidate the function of K. kingae genes that exhibit differential distribution among the associated clinical conditions and, particularly, the potential role of the absence of the *frpC* gene in the transition from asymptomatic carriage to bacterial endocarditis.

No cluster of genes was found to be statistically enriched in the genomes of isolates that caused invasive infection (see Table S1 in the supplemental material). This again supports the possibility that the result of K. kingae infection is not determined by bacterial genetics. However, six genes were statistically enriched in endocarditis-causing strains (see Table S2). It should be mentioned that this list must be carefully examined, because some genes are enriched only as a result of the clustering algorithm and actually are represented in isolates from all conditions. Another point is that none of the genes on this list is found exclusively in all endocarditis-causing isolates, and thus the tissue tropism cannot be fully explained by genetic factors. The functional analysis of five of these genes does not point to any of them having a function that may explain their distribution, such as functions related to virulence, tissue preferences, or biofilm formation. Only *icmT*, an isoprenylcysteine carboxyl methyltransferase, was shown to have an essential role in host cell pore formation, as mutants of this gene in Legionella pneumophila were affected regarding host cell lysis (29). Further investigation of molecular mechanisms and sequencing of additional clinical K. kingae isolate genomes may shed more light on the roles of these genes.

Kingella kingae is a common pathogen of early childhood, causing a variety of invasive diseases affecting the skeletal system and the endocardium. The present study results might contribute to identifying novel targets for a future protective vaccine.

## MATERIALS AND METHODS

### Source of the study strains.

The clinical microbiology laboratory of the Soroka University Medical Center in southern Israel has been a pioneer in the research on K. kingae and its diseases (30) and, over the past 3 decades, has isolated the organism from >150 infected patients and >500 asymptomatic carriers. In addition, the laboratory has gathered invasive K. kingae isolates from other regions of the country and international strains from Europe and North America (15, 16, 31). K. kingae strains derived from patients with endocarditis and other invasive infections that had been collected at the Shaare Zedek Medical Center in Jerusalem, Israel, were also available for the study.

To achieve a representative sample of the K. kingae population, a strain collection was assembled to include strains with a wide variety of PFGE STs that had been isolated between the 1960s and 2021 from individuals with a variety of invasive infections living in different geographic locations, as well as oropharyngeal strains from epidemiologically unrelated carriers (15, 16, 31). All strains have been kept frozen at −80°C in 15% glycerol-containing Mueller-Hinton medium since isolation.

### Clinical conditions associated with the K. kingae isolates.

For the purposes of the data analysis, the clinical conditions associated with the isolates were classified into two broad categories, namely, asymptomatic oropharyngeal carriage and invasive diseases. The latter group comprised bacteremia with no focal infection (occult bacteremia), skeletal system infections (including septic arthritis and osteomyelitis), and bacterial endocarditis. Isolates recovered from blood cultures drawn from patients with focal diseases such as septic arthritis or endocarditis were allocated to the skeletal system or the endocarditis category, respectively, and not to the bacteremia group.

### DNA extraction and WGS.

Genomic DNA was extracted from pure cultures using a DNeasy tissue kit (Qiagen, Germantown, MD) according to the manufacturer’s instructions. DNA libraries were prepared using the Nextera XT kit (Illumina, San Diego, CA) according to the manufacturer’s protocol and were sequenced with 150-bp single-end reads using an Illumina NextSeq platform.

### Bioinformatic analysis.

Raw sequencing reads were trimmed and filtered using Cutadapt, and filtered reads were used for *de novo* assembly with SPAdes v3.13.1 (32). Assemblies were evaluated with QUAST (33). Sequencing and assembly statistics are described in Table S4 in the supplemental material. Each isolate’s assembly was checked for contamination using Kraken v2 (34), and genes were predicted and annotated using Prokka v1.14.6 (35). The presence of plasmids, antimicrobial resistance genes, and virulence genes was predicted using the PlasmidFinder, ResFinder, and Virulence Factor Database (VFDB) databases, respectively.

Pangenomes and core genomes were determined using Roary v3.13.0 (36). Phylogenetic relationships between genomes were assessed by aligning the core genome sequences using MAFFT v7.475 (37), and approximately maximum likelihood phylogenetic trees were built using FastTree v2.1.11 (38), with 1,000 resamples of the Shimodaira-Hasegawa test. MLST of all isolates was performed with the *de novo* assemblies using MLST software (https://cge.food.dtu.dk/services/MLST/). Isolates exhibiting previously undescribed allele combinations were given new ST numbers and deposited in the Institut Pasteur database (http://bigsdb.pasteur.fr/kingella). cgMLST was performed using chewBBACA (39).

### STCs.

Isolates were grouped into STCs if they differed at no more than 1 locus from at least one other member of the group. Founder genotypes of STCs were defined as the ST of the STC with the highest number of neighboring STs (single-locus variants). Early in the research, it became apparent that STC-23 and STC-25 were overrepresented among endocarditis-associated strains. The founder ST of STC-23 and that of STC-25 are closely related, differing at only 2 loci and, for the purposes of the analysis, we gathered these two STCs into a single STC and named it STC-23/25. The resulting STC-23/25 included ST-21, -22, -23, -24, -25, -26, -44, -55, -56, and -62. To compare the genomic content of STC-23/25 endocarditis-associated strains with that of strains derived from individuals with other conditions, the study population was enriched with additional STC-23/25 organisms isolated from healthy carriers, patients with bacteremia, and children with skeletal infections.

### Statistical analysis.

Gene contents in isolates related to the different clinical conditions were compared using the chi-square test, and *P* values were adjusted for multiple tests with the Bonferroni correction. *P* values of <0.05 were considered significant for all calculations. One-way ANOVA was employed to find genes whose distribution showed significant differences between the various clinical condition groups. Statistical analysis and data visualization were conducted in R v4.0.3 using the ggplot2 and pheatmap packages.

### Ethics approval.

Ethics approval was obtained from the local institutional review board (Helsinki Ethics Committee) (approval number 0511-20-SZMC).

### Data availability.

The raw sequencing data for K. kingae isolates reported in this study have been deposited in the NCBI Sequence Read Archive (SRA) under BioProject accession number PRJNA891445. The genome assemblies have been deposited in the BIGSdb-Pasteur database (https://bigsdb.pasteur.fr/kingella).

## Supplementary Material

Reviewer comments
