# Supplementary material for: Whole-Genome Sequencing Reveals Differences among Kingella kingae Strains from Carriers and Patients with Invasive Infections
Source: Microbiol Spectr. 2023 May 17;11(3):e03895-22. doi: 10.1128/spectrum.03895-22 (PMC10269580; doi:10.1128/spectrum.03895-22)
Supplement: Supplemental file 5 — Fig. S1 to S3. Download spectrum.03895-22-s0001.pdf, PDF file, 0.7 MB [file spectrum.03895-22-s0001.pdf]

Figure S1

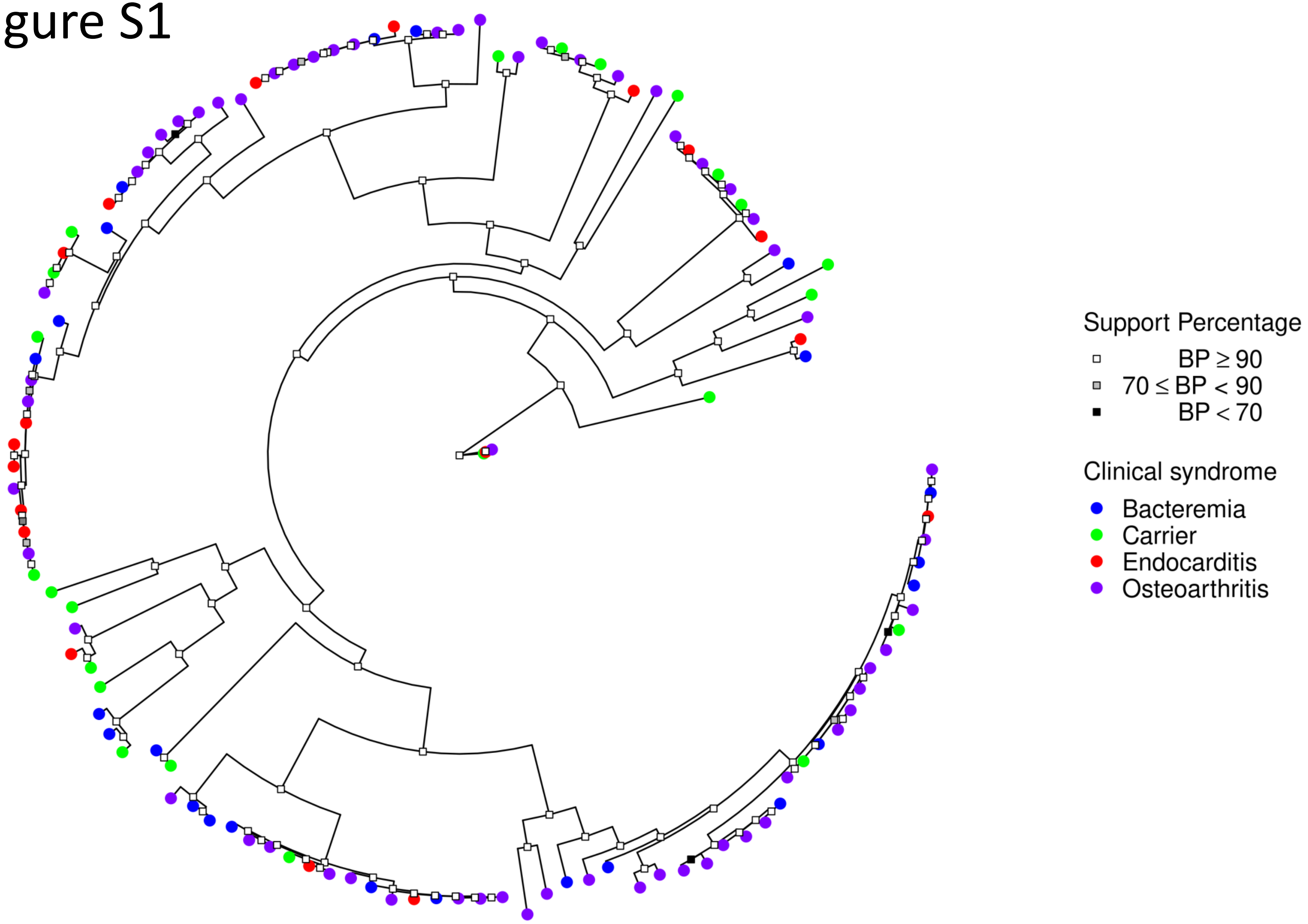

Fig S1. An approximate maximum likelihood phylogenetic tree of all 125 isolates based on nucleotide alignment of the core genomes. Edge colors indicate clinical outcome of the isolates. Node colors indicate Shimodaira-Hasegawa test support fraction.

Figure S2

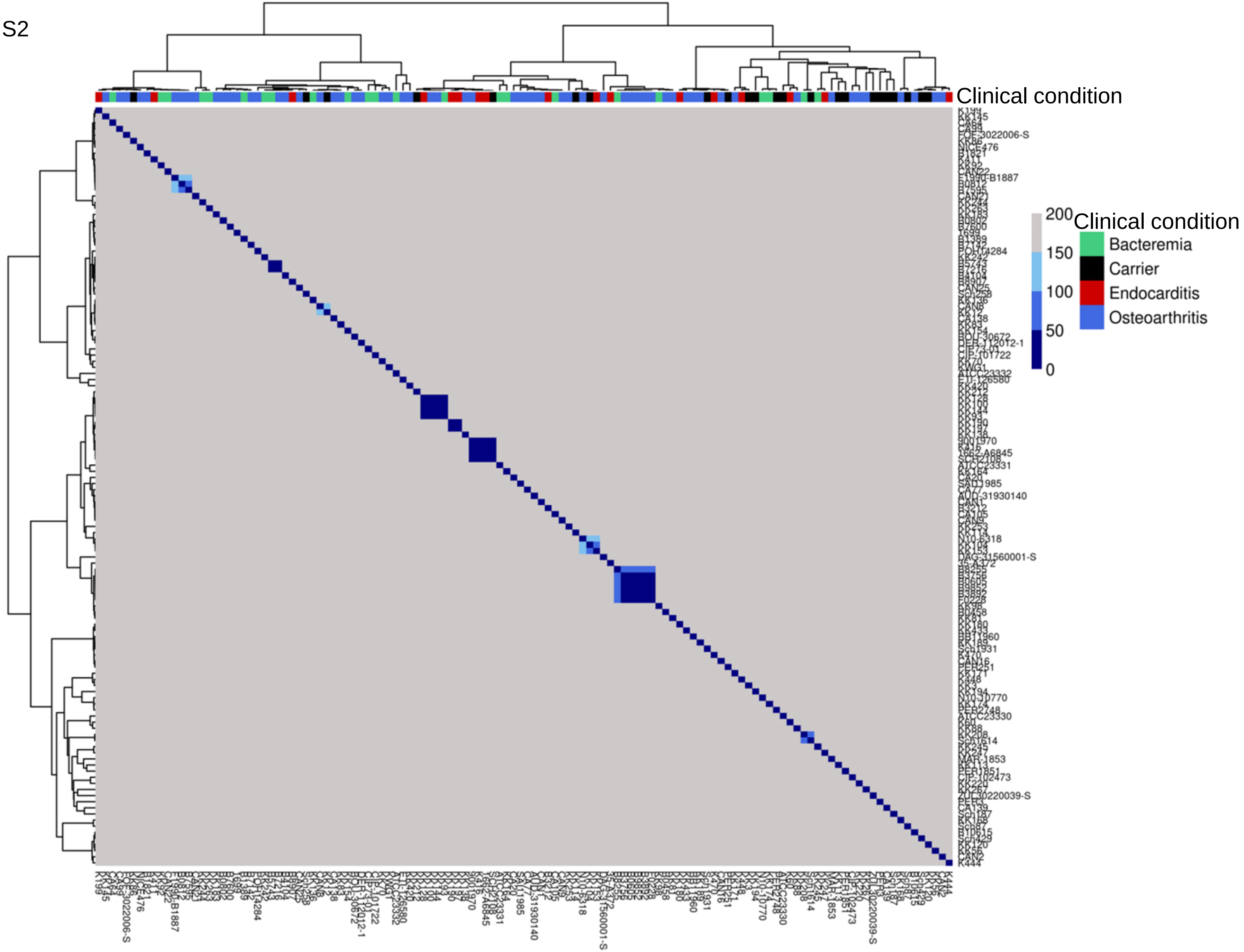

Fig S2. Clustered heatmap showing the pairwise SNP distance between any two isolates.

Figure S3

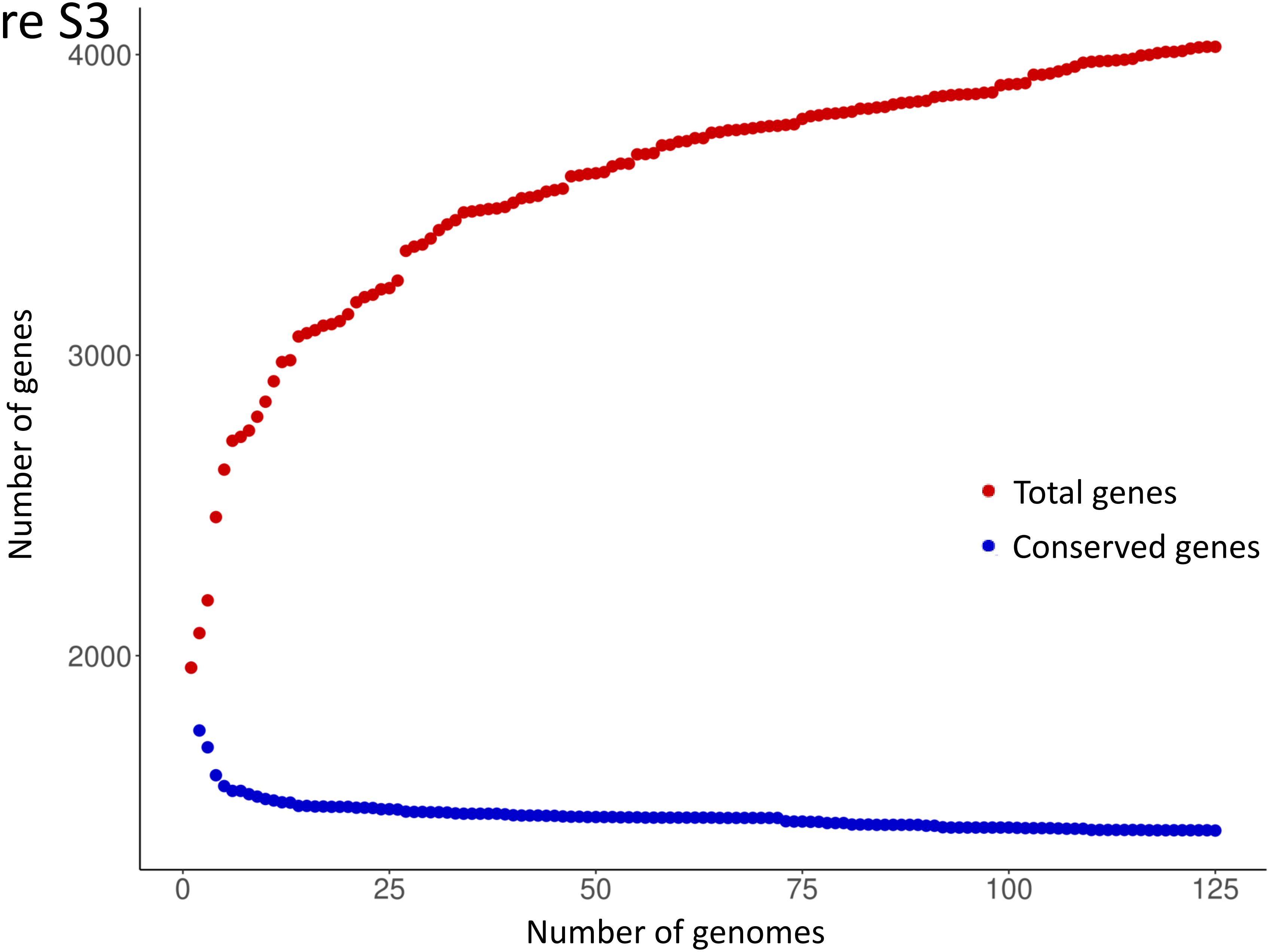

Fig S3. The effect of the number of genomes included in the pangenome on the total and core gene counts.
