# Supplementary material for: Whole-Genome Sequencing Reveals Differences among Kingella kingae Strains from Carriers and Patients with Invasive Infections
Source: Microbiol Spectr. 2023 May 17;11(3):e03895-22. doi: 10.1128/spectrum.03895-22 (PMC10269580; doi:10.1128/spectrum.03895-22)
Supplement: Supplemental file 1 — Table S1. Download spectrum.03895-22-s0002.docx, DOCX file, 0.02 MB [file spectrum.03895-22-s0002.docx]

**Table S1**. Genes enriched in *K. kingae* isolates associated with invasive disease compared to carried organisms.

| **Pangenome orthogroup ID** | **Best BLAST hit** | **Endocarditis**  **n=18** | **Osteoarthritis**  **n=61** | **Bacteremia**  **n=23** | **Carrier**  **n=23** | **Enriched in invasive isolates (p-value)** | **Adjusted  p-value** |
| --- | --- | --- | --- | --- | --- | --- | --- |
| group_2550 | Virulence-associated protein B | 5 | 29 | 14 | 2 | 0.015321 | 0.733 |
| pilY1_1 | VWA domain-containing protein | 5 | 28 | 14 | 2 | 0.015337 | 0.733 |
| group_2224 | TrbC/VirB2 family protein | 4 | 28 | 13 | 2 | 0.016116 | 0.733 |
| group_2526 | PilC2 | 2 | 13 | 7 | 0 | 0.025051 | 0.733 |
| pcp | Pyroglutamyl-peptidase I, partial | 4 | 26 | 13 | 2 | 0.025916 | 0.733 |
| group_2601 | Protein of unknown function | 4 | 26 | 13 | 2 | 0.025916 | 0.733 |
| group_847 | Putative prophage CPS-53 integrase | 4 | 26 | 13 | 2 | 0.025916 | 0.733 |
| group_848 | Phage integrase family site-specific recombinase | 4 | 26 | 13 | 2 | 0.025916 | 0.733 |
| group_1442 | Hypothetical protein | 4 | 27 | 13 | 2 | 0.02608 | 0.733 |
| group_1781 | Phage virion morphogenesis protein | 4 | 27 | 13 | 2 | 0.02608 | 0.733 |
| group_2207 | Protein of unknown function | 4 | 27 | 13 | 2 | 0.02608 | 0.733 |
| virB4 | VirB4 family type IV secretion/conjugal transfer ATPase | 4 | 27 | 13 | 2 | 0.02608 | 0.733 |
| group_2226 | Type IV secretion system protein | 4 | 27 | 13 | 2 | 0.02608 | 0.733 |
| group_2257 | Uncharacterized protein | 4 | 27 | 13 | 2 | 0.02608 | 0.733 |
| virB1_1 | Uncharacterized protein | 4 | 27 | 13 | 2 | 0.02608 | 0.733 |
| group_2521 | AlpA family phage regulatory protein | 4 | 27 | 13 | 2 | 0.02608 | 0.733 |
| rimI_2 | GNAT family N-acetyltransferase | 4 | 27 | 13 | 2 | 0.02608 | 0.733 |
| group_2547 | Hypothetical protein | 4 | 27 | 13 | 2 | 0.02608 | 0.733 |
| group_2549 | Uncharacterized protein | 4 | 27 | 13 | 2 | 0.02608 | 0.733 |
| group_2551 | Hypothetical protein | 4 | 27 | 13 | 2 | 0.02608 | 0.733 |
| traL | Hypothetical protein | 4 | 27 | 13 | 2 | 0.02608 | 0.733 |
| group_2574 | Hypothetical protein | 4 | 27 | 13 | 2 | 0.02608 | 0.733 |
| virB9 | TrbG/VirB9 family P-type conjugative transfer protein | 4 | 27 | 13 | 2 | 0.02608 | 0.733 |
| group_2576 | TrbI/VirB10 family protein | 4 | 27 | 13 | 2 | 0.02608 | 0.733 |
| group_2579 | Uncharacterized protein | 4 | 27 | 13 | 2 | 0.02608 | 0.733 |
| group_2583 | Uncharacterized protein | 4 | 27 | 13 | 2 | 0.02608 | 0.733 |
| group_2584 | Hypothetical protein | 4 | 27 | 13 | 2 | 0.02608 | 0.733 |
| group_2586 | Hypothetical protein | 4 | 27 | 13 | 2 | 0.02608 | 0.733 |
| group_2590 | Hypothetical protein | 4 | 27 | 13 | 2 | 0.02608 | 0.733 |
| group_2591 | DUF262 domain-containing protein | 4 | 27 | 13 | 2 | 0.02608 | 0.733 |
| group_2592 | DUF262 domain-containing HNH endonuclease family protein | 4 | 27 | 13 | 2 | 0.02608 | 0.733 |
| group_2593 | Uncharacterized protein | 4 | 27 | 13 | 2 | 0.02608 | 0.733 |
| group_2598 | KilA-N domain-containing protein | 4 | 27 | 13 | 2 | 0.02608 | 0.733 |
| group_2599 | Hypothetical protein | 4 | 27 | 13 | 2 | 0.02608 | 0.733 |
| group_1785 | Hypothetical protein | 4 | 25 | 13 | 2 | 0.026163 | 0.733 |
| group_2254 | Hypothetical protein | 3 | 27 | 12 | 2 | 0.026163 | 0.733 |
| virB3 | VirB3 family type IV secretion system protein | 4 | 25 | 13 | 2 | 0.026163 | 0.733 |
| group_1767 | Protein of unknown function | 2 | 20 | 9 | 1 | 0.029341 | 0.800 |
| group_275 | Uncharacterized protein | 3 | 25 | 11 | 2 | 0.042777 | 1.000 |
| group_1078 | DNA ligase (fragment) | 2 | 25 | 11 | 2 | 0.043037 | 1.000 |
| group_2223 | Conserved exported protein of unknown function | 2 | 18 | 9 | 1 | 0.047703 | 1.000 |
| group_2548 | Uncharacterized protein | 2 | 17 | 9 | 1 | 0.048094 | 1.000 |
| group_578 | Porphobilinogen synthase | 2 | 17 | 9 | 1 | 0.048094 | 1.000 |
