# Supplementary material for: Whole-Genome Sequencing Reveals Differences among Kingella kingae Strains from Carriers and Patients with Invasive Infections
Source: Microbiol Spectr. 2023 May 17;11(3):e03895-22. doi: 10.1128/spectrum.03895-22 (PMC10269580; doi:10.1128/spectrum.03895-22)
Supplement: Supplemental file 2 — Table S2. Download spectrum.03895-22-s0003.docx, DOCX file, 0.02 MB [file spectrum.03895-22-s0003.docx]

**Table S2**. Genes enriched in *K. kingae* isolates associated with endocarditis compared to other isolates.

| **Pangenome ID** | **Best BLAST hit** | **Description** | **Endocarditis**  **n=18** | **Osteoarthritis**  **n=61** | **Bacteremia**  **n=23** | **Carrier**  **n=23** | **Enriched in endocarditis isolates (p-value)** | **Adjusted p-value** |
| --- | --- | --- | --- | --- | --- | --- | --- | --- |
| group_633 | PAS sensor domain-containing protein | PAS domain | 9 | 0 | 0 | 2 | 5.87E-07 | 0.0006 |
| group_570 | Elongation factor Tu | This protein promotes the GTP-dependent binding of aminoacyl-tRNA to the A-site of ribosomes during protein biosynthesis | 13 | 4 | 3 | 6 | 1.11E-05 | 0.0038 |
| group_252 | Eco57I restriction endonuclease | T5orf172 | 13 | 4 | 3 | 5 | 1.11E-05 | 0.0038 |
| group_555 | Isoprenylcysteine carboxyl methyltransferase | Isoprenylcysteine carboxyl methyltransferase (ICMT) family | 13 | 5 | 3 | 9 | 2.24E-05 | 0.0058 |
| group_2475 | Hypothetical protein | - | 6 | 0 | 0 | 4 | 9.57E-05 | 0.0188 |
| hisG_2 | ATP phosphoribosyltransferase | ATP phosphoribosyltransferase | 11 | 4 | 3 | 5 | 1.09E-04 | 0.0188 |
| group_113 | Modification methylase DpnIIA | D12 class N6 adenine-specific DNA methyltransferase | 5 | 0 | 0 | 1 | 5.61E-04 | 0.0684 |
| group_1864 | DUF3861 family protein | Domain of unknown function with PDB structure (DUF3861) | 11 | 7 | 3 | 7 | 5.76E-04 | 0.0684 |
| group_1225 | *No BLAST hits* | - | 9 | 5 | 3 | 3 | 5.94E-04 | 0.0684 |
| group_759 | Hypothetical protein | - | 10 | 6 | 3 | 8 | 9.87E-04 | 0.0970 |
| group_2435 | *No BLAST hits* | - | 8 | 4 | 3 | 5 | 1.03E-03 | 0.0970 |
| group_1361 | Sulfite exporter TauE/SafE family protein | Membrane transporter protein | 14 | 11 | 8 | 14 | 1.71E-03 | 0.1379 |
| group_1929 | Hypothetical protein | - | 8 | 5 | 3 | 8 | 1.73E-03 | 0.1379 |
| group_105 | DNA adenine methylase | D12 class N6 adenine-specific DNA methyltransferase | 9 | 7 | 2 | 7 | 2.74E-03 | 0.1887 |
| group_700 | Potential acrAB operon repressor | MAATS-type transcriptional repressor, C-terminal region | 9 | 4 | 5 | 5 | 2.74E-03 | 0.1887 |
| group_1666 | Tryptophan synthase subunit beta | The beta subunit is responsible for the synthesis of L- tryptophan from indole and L-serine | 13 | 12 | 6 | 14 | 2.92E-03 | 0.1887 |
| group_2505 | Protein of unknown function | - | 4 | 0 | 0 | 2 | 3.42E-03 | 0.1887 |
| group_2642 | Anthranilate synthase component II | - | 3 | 0 | 0 | 0 | 3.42E-03 | 0.1887 |
| group_1308 | Hypothetical protein HMPREF0476_1255 | - | 9 | 9 | 3 | 9 | 3.46E-03 | 0.1887 |
| group_286 | *Neisseria meningitidis* TspB protein | *Neisseria meningitidis* TspB protein | 4 | 1 | 1 | 2 | 6.29E-03 | 0.3103 |
| group_2775 | Hypothetical protein HMPREF0476_0055 | - | 4 | 1 | 1 | 0 | 6.29E-03 | 0.3103 |
| group_43 | Uncharacterized protein | - | 4 | 1 | 0 | 2 | 1.20E-02 | 0.4973 |
| group_112 | Modification methylase DpnIIA | D12 class N6 adenine-specific DNA methyltransferase | 4 | 1 | 0 | 0 | 1.20E-02 | 0.4973 |
| group_187 | Conserved protein of unknown function | Transposase, mutator family of *Pasteurellaceae* UniRef RepID F4H9B5_GALAU | 4 | 0 | 1 | 0 | 1.20E-02 | 0.4973 |
| group_188 | Conserved protein of unknown function | Transposase, mutator family of *Pasteurellaceae* UniRef RepID F4H9B5_GALAU | 4 | 0 | 1 | 0 | 1.20E-02 | 0.4973 |
| group_1199 | Choline binding protein C | PFAM transposase, IS4 family protein | 3 | 1 | 1 | 1 | 2.64E-02 | 1.0000 |
| group_2324 | Hypothetical protein HMPREF0476_0036 | - | 4 | 1 | 1 | 0 | 2.64E-02 | 1.0000 |
| group_24 | Iron-regulated protein FrpC (modular protein) | COG2931, RTX toxins and related Ca2 -binding proteins | 0 | 18 | 9 | 4 | 4.33E-02 | 1.0000 |
