# Supplementary material for: Whole-Genome Sequencing Reveals Differences among Kingella kingae Strains from Carriers and Patients with Invasive Infections
Source: Microbiol Spectr. 2023 May 17;11(3):e03895-22. doi: 10.1128/spectrum.03895-22 (PMC10269580; doi:10.1128/spectrum.03895-22)
Supplement: Supplemental file 3 — Table S3. Download spectrum.03895-22-s0004.docx, DOCX file, 0.03 MB [file spectrum.03895-22-s0004.docx]

**Table S3.** Genes enriched in *K. kingae* isolates belonging to STC-23/25 associated with endocarditis compared to STC-23/25 isolates derived from other clinical conditions.

| **Pangenome ID** | **Best BLAST hit** | **Description** | **Endocarditis (n=8)** | **Non-endocarditis (n=25)** | **p-value** | **Adjusted p-value** |
| --- | --- | --- | --- | --- | --- | --- |
| group_131 | Hypothetical protein | Phage Mu protein F like protein | 5 | 1 | 0.011 | 1 |
| group_134 | Phage head morphogenesis protein | Phage Mu protein F like protein | 5 | 1 | 0.011 | 1 |
| group_1720 | *No BLAST hits* | - | 5 | 1 | 0.011 | 1 |
| group_1164 | Hypothetical protein | - | 5 | 2 | 0.027 | 1 |
| group_1139 | Hypothetical protein | Maltose O-acetyltransferase activity | 5 | 2 | 0.027 | 1 |
| group_2456 | Hypothetical protein | - | 5 | 2 | 0.027 | 1 |
| group_1001 | Hypothetical protein | Sister chromatid segregation | 6 | 3 | 0.041 | 1 |
| group_2177 | *No BLAST hits* | - | 6 | 3 | 0.041 | 1 |
| group_2081 | DUF4224 domain-containing protein | Domain of unknown function (DUF4224) | 6 | 3 | 0.041 | 1 |
| group_1071 | Hypothetical protein | - | 6 | 4 | 0.055 | 1 |
| group_1188 | Helix-turn-helix domain-containing protein | Transcriptional regulator | 6 | 4 | 0.055 | 1 |
| group_1572 | Hypothetical protein | - | 6 | 4 | 0.055 | 1 |
| group_2454 | Hypothetical protein | - | 6 | 4 | 0.055 | 1 |
| group_2455 | DUF1870 family protein | Domain of unknown function (DUF1870) | 6 | 4 | 0.055 | 1 |
| dnaA_2 | Hypothetical protein | IstB-like ATP binding protein | 6 | 4 | 0.055 | 1 |
| group_1415 | Hypothetical protein E5V35_00770 | TIGRFAM phage protein, HK97 gp10 family | 6 | 4 | 0.055 | 1 |
| group_1570 | Hypothetical protein | Helix-turn-helix domain | 6 | 4 | 0.055 | 1 |
| group_1576 | DUF3168 domain-containing protein | Protein of unknown function (DUF3168) | 6 | 4 | 0.055 | 1 |
| group_1972 | Hypothetical protein | Bacteriophage Lambda NinG protein | 6 | 4 | 0.055 | 1 |
| group_1973 | Hypothetical protein | NinB protein | 6 | 4 | 0.055 | 1 |
| group_2082 | Hypothetical protein D8B42_09245 | - | 6 | 4 | 0.055 | 1 |
| group_2083 | Hypothetical protein D8B42_00535 | - | 6 | 4 | 0.055 | 1 |
| group_2426 | Hypothetical protein | - | 6 | 4 | 0.055 | 1 |
| group_1108 | lysozyme | lysozyme | 6 | 4 | 0.055 | 1 |
| group_1641 | Hypothetical protein | Bacteriophage holin family, superfamily II-like | 6 | 4 | 0.055 | 1 |
| group_2084 | Hypothetical protein | - | 6 | 4 | 0.055 | 1 |
| group_2453 | Tyrosine-type recombinase/integrase | Belongs to the 'phage' integrase family | 6 | 4 | 0.055 | 1 |
| group_1575 | Hypothetical protein | - | 6 | 4 | 0.055 | 1 |
| group_1976 | Phage major capsid protein | Phage capsid family | 6 | 4 | 0.055 | 1 |
| group_1573 | HNH endonuclease | HNH nucleases | 6 | 4 | 0.055 | 1 |
| group_1574 | Hypothetical protein | - | 6 | 4 | 0.055 | 1 |
| group_1974 | SRPBCC family protein | - | 6 | 4 | 0.055 | 1 |
| clpP_2 | Clp protease ClpP | Belongs to the peptidase S14 family | 6 | 4 | 0.055 | 1 |
| group_1977 | Head-tail adaptor protein | Phage head-tail joining protein | 6 | 4 | 0.055 | 1 |
| group_2422 | Hypothetical protein | Terminase small subunit | 6 | 4 | 0.055 | 1 |
| group_2423 | Terminase large subunit | Phage Terminase | 6 | 4 | 0.055 | 1 |
| group_2424 | Phage portal protein | Phage portal protein | 6 | 4 | 0.055 | 1 |
| group_2427 | Unnamed protein product | - | 6 | 4 | 0.055 | 1 |
| group_1978 | Hypothetical protein | - | 6 | 4 | 0.055 | 1 |
| group_2425 | Phage gp6-like head-tail connector protein | Phage gp6-like head-tail connector protein | 6 | 4 | 0.055 | 1 |
| group_1378 | DUF2335 domain-containing protein | Predicted membrane protein (DUF2335) | 6 | 4 | 0.055 | 1 |
| group_2420 | Hypothetical protein | - | 6 | 4 | 0.055 | 1 |
| group_1566 | Hypothetical protein | - | 6 | 4 | 0.055 | 1 |
| group_681 | Hypothetical protein | - | 6 | 4 | 0.055 | 1 |
| lexA_1 | Helix-turn-helix domain-containing protein | Peptidase S24-like | 6 | 4 | 0.055 | 1 |
| group_510 | Hypothetical protein | - | 6 | 4 | 0.055 | 1 |
| recT | Recombination protein RecT | RecT family | 6 | 4 | 0.055 | 1 |
| group_1069 | DUF4041 domain-containing protein | T5orf172 | 6 | 4 | 0.055 | 1 |
| group_2421 | Helix-turn-helix transcriptional regulator | Protein conserved in bacteria | 4 | 2 | 0.060 | 1 |
| group_141 | Phage tail tape measure protein, partial | Phage-related minor tail protein | 4 | 2 | 0.060 | 1 |
| group_2754 | Hypothetical protein HMPREF0476_0055 | - | 4 | 2 | 0.060 | 1 |
| group_875 | DNA-binding protein | Cytotoxic translational repressor of toxin-antitoxin stability system | 3 | 1 | 0.070 | 1 |
| group_212 | Hypothetical protein | - | 3 | 1 | 0.070 | 1 |
| group_2485 | Protein of unknown function | - | 3 | 1 | 0.070 | 1 |
| group_1047 | PIN domain-containing protein | PIN domain | 2 | 0 | 0.076 | 1 |
| group_1748 | Hypothetical protein | - | 2 | 0 | 0.076 | 1 |
| group_219 | Hypothetical protein | - | 2 | 0 | 0.076 | 1 |
| group_609 | Toxin-activating lysine-acyltransferase | Involved in fatty acylation of the protoxin (CyaA) at two internal lysine residues, thereby converting it to the active toxin. The acyl donor is ACP | 2 | 0 | 0.076 | 1 |
| ssb_2 | Single-stranded DNA-binding protein | Plays an important role in DNA replication, recombination and repair. Binds to ssDNA and to an array of partner proteins to recruit them to their sites of action during DNA metabolism | 5 | 3 | 0.084 | 1 |
| group_958 | KilA-N domain-containing protein | KilA-N | 5 | 3 | 0.084 | 1 |
