# Supplementary material for: Whole-Genome Sequencing Reveals Differences among Kingella kingae Strains from Carriers and Patients with Invasive Infections
Source: Microbiol Spectr. 2023 May 17;11(3):e03895-22. doi: 10.1128/spectrum.03895-22 (PMC10269580; doi:10.1128/spectrum.03895-22)
Supplement: Supplemental file 4 — Table S4. Download spectrum.03895-22-s0005.docx, DOCX file, 0.04 MB [file spectrum.03895-22-s0005.docx]

**Table S4**. Summary statistics for the sequencing and assembly of the 125 isolates sequenced in this study.

| Isolate | # contigs | Total length | N50 | # total reads | Mapped (%) | Avg. coverage depth | # N's per 100 kbp |
| --- | --- | --- | --- | --- | --- | --- | --- |
| 1662-A6845 | 82 | 1,969,098 | 64,958 | 5,999,934 | 98.84 | 377 | 4.93 |
| 1699 | 112 | 1,997,408 | 43,837 | 1,626,742 | 99.06 | 101 | 4.91 |
| 35-A372 | 89 | 1,961,810 | 71,184 | 4,191,129 | 99.17 | 273 | 5.05 |
| 9001970 | 99 | 1,971,261 | 40,308 | 1,209,971 | 98.54 | 72 | 0 |
| ATCC23330 | 92 | 1,923,134 | 54,617 | 1,388,867 | 98.45 | 89 | 0 |
| ATCC23331 | 81 | 1,961,471 | 78,662 | 4,703,654 | 99.31 | 311 | 4.95 |
| ATCC23332 | 102 | 1,962,944 | 43,836 | 1,253,317 | 98.83 | 78 | 5.04 |
| AUD_319301 | 93 | 1,954,055 | 64,990 | 2,034,261 | 99.24 | 121 | 5.12 |
| B0458 | 83 | 1,954,511 | 68,950 | 1,721,539 | 98.72 | 110 | 9.67 |
| B0605 | 108 | 2,000,005 | 42,281 | 974,137 | 98.9 | 60 | 0 |
| B0802 | 109 | 1,997,515 | 49,902 | 1,481,841 | 98.62 | 92 | 0 |
| B0812 | 119 | 2,021,600 | 55,444 | 1,094,790 | 98.97 | 68 | 0 |
| B10615 | 96 | 1,978,447 | 56,201 | 1,179,681 | 98.37 | 73 | 5.05 |
| B1389 | 110 | 1,998,152 | 47,542 | 1,285,550 | 98.6 | 80 | 9.91 |
| B1821 | 115 | 2,017,675 | 45,182 | 1,296,883 | 99.08 | 81 | 0 |
| B3212 | 93 | 1,996,161 | 64,990 | 6,414,023 | 99.3 | 400 | 9.92 |
| B3756 | 94 | 1,998,810 | 68,993 | 1,088,000 | 98.74 | 69 | 9.91 |
| B3892 | 92 | 1,998,948 | 68,897 | 1,770,914 | 98.86 | 109 | 0 |
| B4104 | 137 | 2,032,689 | 43,924 | 1,641,527 | 98.63 | 97 | 14.56 |
| B5743 | 116 | 2,013,770 | 47,542 | 1,919,944 | 99.29 | 120 | 4.97 |
| B6249 | 128 | 1,913,138 | 37,901 | 1,621,972 | 82.84 | 122 | 5.02 |
| B7142 | 118 | 1,998,349 | 47,542 | 1,505,275 | 98.69 | 93 | 9.96 |
| B7216 | 119 | 2,012,706 | 47,542 | 1,525,713 | 99.07 | 95 | 0 |
| B7595 | 120 | 2,023,917 | 49,257 | 1,187,958 | 98.95 | 74 | 0 |
| B7600 | 97 | 1,959,256 | 50,725 | 1,721,874 | 98.78 | 110 | 4.95 |
| B8255 | 105 | 2,032,196 | 67,046 | 2,103,205 | 98.79 | 124 | 4.82 |
| B8907 | 112 | 2,010,185 | 47,542 | 9,068,104 | 99.01 | 543 | 0 |
| B9852 | 97 | 1,999,263 | 68,897 | 1,488,191 | 98.76 | 93 | 9.9 |
| BB11960 | 82 | 1,942,926 | 66,658 | 1,468,662 | 99.04 | 94 | 9.98 |
| BOU_30672 | 98 | 1,955,039 | 50,725 | 1,484,580 | 98.9 | 93 | 0 |
| CA105 | 83 | 1,949,678 | 84,562 | 1,936,755 | 99.19 | 119 | 5.08 |
| CA138 | 97 | 1,953,888 | 45,730 | 1,657,426 | 99.23 | 100 | 0 |
| CA139 | 82 | 1,930,373 | 57,092 | 1,791,712 | 98.61 | 114 | 5.08 |
| CA20 | 85 | 1,952,970 | 84,615 | 7,512,305 | 99.58 | 488 | 0 |
| CA64 | 118 | 2,022,377 | 44,062 | 1,664,027 | 98.83 | 103 | 0 |
| CA77 | 83 | 1,949,296 | 84,492 | 1,365,613 | 99.07 | 89 | 5.08 |
| CA99 | 109 | 1,976,704 | 51,065 | 1,806,842 | 98.61 | 113 | 0 |
| CAN16 | 83 | 1,942,916 | 61,844 | 1,389,372 | 99.14 | 91 | 10.04 |
| CAN1 | 85 | 1,948,126 | 66,844 | 1,703,033 | 99.1 | 107 | 0 |
| CAN21 | 97 | 1,950,178 | 57,238 | 1,552,665 | 98.93 | 100 | 0 |
| CAN22 | 113 | 1,982,458 | 55,356 | 1,876,753 | 99.07 | 115 | 0 |
| CAN25 | 108 | 1,967,388 | 45,136 | 2,053,265 | 99.34 | 122 | 0 |
| CAN2 | 93 | 1,935,416 | 44,141 | 962,304 | 98.18 | 61 | 0 |
| CAN8 | 100 | 1,955,138 | 47,470 | 1,953,878 | 99.46 | 119 | 0 |
| CAN9 | 89 | 1,949,881 | 65,083 | 1,808,432 | 99.09 | 113 | 5.03 |
| CIP_101722 | 96 | 1,958,475 | 45,137 | 1,364,672 | 99.37 | 83 | 0 |
| CIP_102473 | 90 | 1,984,530 | 41,487 | 1,719,007 | 98.91 | 106 | 4.94 |
| CIP73-01 | 104 | 1,957,059 | 47,576 | 1,595,702 | 99.34 | 101 | 0 |
| DAG_315600 | 89 | 1,917,195 | 70,758 | 1,484,621 | 98.79 | 98 | 10.22 |
| DER_112012 | 101 | 1,954,964 | 45,185 | 1,954,692 | 99.23 | 119 | 0 |
| ETI_126580 | 100 | 1,941,378 | 45,085 | 2,013,368 | 99.02 | 123 | 5 |
| F0228 | 96 | 1,997,805 | 55,864 | 1,209,753 | 98.61 | 74 | 0 |
| F1990_B188 | 134 | 2,046,060 | 38,593 | 1,159,448 | 98.67 | 70 | 0 |
| FOF_302200 | 121 | 2,015,159 | 55,356 | 1,450,407 | 98.56 | 87 | 0 |
| K199 | 111 | 1,974,351 | 44,195 | 1,040,879 | 99.63 | 65 | 0 |
| K411 | 111 | 1,984,594 | 55,356 | 6,251,366 | 98.95 | 394 | 0 |
| K416 | 81 | 1,970,135 | 67,314 | 1,644,607 | 99.25 | 105 | 4.97 |
| K444 | 88 | 1,980,344 | 56,367 | 1,102,394 | 98.91 | 68 | 5.05 |
| K448 | 83 | 1,935,583 | 93,029 | 5,295,475 | 99.09 | 358 | 5.06 |
| K470 | 80 | 1,943,174 | 66,875 | 4,636,960 | 99.31 | 316 | 0 |
| K60 | 87 | 1,935,016 | 66,885 | 1,193,476 | 99.2 | 78 | 5.12 |
| KK100 | 76 | 1,931,538 | 64,958 | 5,195,861 | 99.28 | 349 | 5.07 |
| KK104 | 86 | 1,996,497 | 66,843 | 5,100,318 | 99.53 | 334 | 9.72 |
| KK113 | 88 | 1,979,648 | 44,583 | 1,154,755 | 99.51 | 74 | 0 |
| KK114 | 79 | 1,959,999 | 66,842 | 4,768,805 | 99.28 | 315 | 15.1 |
| KK120 | 80 | 1,933,454 | 67,454 | 1,216,991 | 98.5 | 80 | 0 |
| KK128 | 83 | 1,968,430 | 64,958 | 5,380,425 | 99.2 | 355 | 9.91 |
| KK12 | 101 | 1,954,850 | 50,725 | 1,666,160 | 99.22 | 103 | 0 |
| KK136 | 95 | 1,954,154 | 56,375 | 1,422,170 | 98.98 | 85 | 0 |
| KK138 | 79 | 1,933,191 | 67,314 | 1,519,310 | 98.91 | 99 | 14.95 |
| KK144 | 76 | 1,932,324 | 67,219 | 5,223,957 | 99.2 | 350 | 9.88 |
| KK145 | 109 | 1,974,595 | 44,158 | 1,967,277 | 99.08 | 112 | 0 |
| KK153 | 98 | 2,026,055 | 66,981 | 5,230,119 | 99.52 | 338 | 14.51 |
| KK154 | 116 | 1,965,656 | 36,388 | 2,050,585 | 98.54 | 110 | 0 |
| KK164 | 87 | 1,959,226 | 64,623 | 4,743,889 | 99.48 | 316 | 9.95 |
| KK168 | 85 | 1,951,754 | 67,833 | 1,662,427 | 99.02 | 104 | 14.91 |
| KK171 | 90 | 1,936,297 | 58,256 | 1,604,005 | 98.6 | 100 | 14.93 |
| KK174 | 110 | 1,925,022 | 29,753 | 1,536,138 | 100.42 | 93 | 4.99 |
| KK180 | 76 | 1,944,367 | 66,889 | 7,790,928 | 99.21 | 494 | 0 |
| KK183 | 109 | 1,993,035 | 49,902 | 1,735,318 | 98.78 | 105 | 4.82 |
| KK189 | 80 | 1,943,327 | 58,035 | 1,557,599 | 99.16 | 100 | 5.09 |
| KK190 | 89 | 1,996,633 | 65,133 | 6,392,634 | 98.92 | 411 | 9.77 |
| KK194 | 83 | 1,933,090 | 76,686 | 1,491,535 | 98.67 | 92 | 5.17 |
| KK197 | 89 | 1,997,672 | 65,032 | 5,268,755 | 99.01 | 342 | 4.86 |
| KK208 | 96 | 1,926,351 | 56,748 | 1,694,454 | 98.57 | 100 | 5.09 |
| KK212 | 83 | 1,968,040 | 65,063 | 4,656,854 | 99.38 | 312 | 4.98 |
| KK220 | 103 | 2,005,664 | 39,333 | 1,619,806 | 99.21 | 97 | 4.89 |
| KK242 | 148 | 2,009,279 | 29,472 | 410,223 | 99.55 | 25 | 0 |
| KK244 | 113 | 1,982,613 | 55,368 | 1,430,788 | 98.55 | 88 | 0 |
| KK245 | 112 | 2,078,222 | 51,054 | 1,788,188 | 98.61 | 104 | 4.72 |
| KK247 | 115 | 2,076,169 | 47,590 | 7,373,322 | 99 | 446 | 0 |
| KK253 | 81 | 1,946,123 | 84,480 | 1,729,473 | 99.02 | 109 | 10.07 |
| KK263 | 118 | 1,984,102 | 51,210 | 1,544,544 | 98.99 | 96 | 0 |
| KK267 | 109 | 1,955,503 | 40,686 | 1,711,088 | 98.43 | 98 | 0 |
| KK3 | 84 | 1,935,070 | 67,702 | 1,147,524 | 98.99 | 75 | 10.08 |
| KK420 | 108 | 1,968,753 | 44,878 | 1,377,582 | 98.68 | 86 | 0 |
| KK433 | 84 | 1,942,409 | 55,738 | 1,598,034 | 98.26 | 96 | 4.99 |
| KK56 | 88 | 1,971,199 | 56,201 | 1,281,666 | 98.41 | 79 | 5.07 |
| KK70 | 107 | 1,968,479 | 47,478 | 1,163,848 | 98.91 | 73 | 4.93 |
| KK81 | 110 | 1,955,511 | 46,507 | 2,040,087 | 98.81 | 112 | 5.01 |
| KK83 | 106 | 1,956,419 | 47,557 | 1,974,752 | 99.11 | 115 | 0 |
| KK86 | 132 | 1,985,331 | 33,961 | 1,956,558 | 99.94 | 115 | 0 |
| KK88 | 89 | 1,931,022 | 63,246 | 1,223,405 | 98.53 | 77 | 5.18 |
| KK92 | 123 | 1,981,929 | 36,183 | 1,568,119 | 99.35 | 87 | 0 |
| KK93 | 77 | 1,932,831 | 67,219 | 6,594,971 | 99.23 | 445 | 5.02 |
| KK98 | 102 | 1,952,881 | 43,109 | 1,889,564 | 98.63 | 108 | 10.09 |
| KWG1 | 106 | 2,029,079 | 55,212 | 1,424,232 | 98.68 | 83 | 0 |
| MAR_1853 | 90 | 1,977,948 | 66,884 | 1,679,348 | 99.01 | 98 | 0 |
| N10-10770 | 71 | 1,922,756 | 91,286 | 1,421,518 | 99.15 | 94 | 0 |
| N10-6318 | 79 | 1,958,728 | 66,842 | 6,513,604 | 99.2 | 428 | 9.85 |
| NICE476 | 110 | 1,977,874 | 57,953 | 1,441,452 | 98.8 | 87 | 4.95 |
| PER1851 | 80 | 1,976,958 | 58,036 | 1,360,828 | 98.84 | 84 | 0 |
| PER251 | 76 | 1,945,150 | 67,010 | 1,664,521 | 98.85 | 101 | 0 |
| PER2748 | 72 | 1,892,537 | 76,250 | 1,726,704 | 98.98 | 113 | 5.07 |
| PER3 | 106 | 2,013,367 | 54,581 | 1,738,973 | 99.07 | 102 | 19.42 |
| POH14284 | 116 | 1,967,280 | 34,183 | 1,580,919 | 99.34 | 88 | 0 |
| SAI11985 | 84 | 1,949,554 | 65,059 | 1,558,133 | 98.54 | 97 | 4.98 |
| Sch1614 | 144 | 1,927,945 | 27,532 | 1,407,214 | 99.76 | 81 | 5.03 |
| Sch187 | 96 | 1,994,972 | 60,987 | 1,741,598 | 98.01 | 102 | 4.96 |
| Sch1931 | 80 | 1,944,972 | 66,920 | 1,807,550 | 98.72 | 107 | 0 |
| SCH2108 | 91 | 1,971,316 | 64,958 | 6,407,750 | 98.92 | 412 | 0 |
| Sch258 | 102 | 1,970,693 | 50,725 | 1,655,988 | 98.58 | 99 | 0 |
| Sch429 | 86 | 1,972,432 | 43,555 | 1,795,172 | 98.23 | 110 | 4.97 |
| Sch87 | 109 | 1,948,141 | 36,874 | 456,650 | 99.02 | 28 | 10.27 |
| ZUL3022003 | 100 | 1,931,300 | 39,192 | 897,610 | 98.64 | 57 | 5.02 |
